# Repeated FRAP of the actin-binding protein CapG in the cell nucleus—a functional assay for EGF signaling in the single live breast cancer cell

**DOI:** 10.1038/s41598-024-73887-7

**Published:** 2024-10-05

**Authors:** M. K. Fernandez, M. Sinha, R. Kühnemuth, M. Renz

**Affiliations:** 1 Stanford University, Stanford, USA; 2 Heinrich-Heine University Düsseldorf, Düsseldorf, Germany; 3 Department of Gynecology With Center for Oncological Surgery, Charité – Universitätsmedizin Berlin, Mittelallee 9, 13353 Berlin, Germany

**Keywords:** Cancer, Cell biology, Nuclear transport

## Abstract

Compartmentalization and differential distribution of proteins within a cell maintain cellular function and viability. CapG is a gelsolin-related actin-binding protein that distributes in steady state diffusively throughout cytoplasm and cell nucleus. To detect changes in CapG’s nucleocytoplasmic shuttling in response to external stimuli on the single cell level, we established repeated FRAP experiments of one and the same breast cancer cell. With this experimental set up, we found that ATP-depletion reversibly decreased CapG’s shuttling into the cell nucleus. The addition of epidermal growth factor (EGF) increased CapG’s nuclear shuttling within minutes. Serum-starvation doubled the number of breast cancer cells from 40 to 80% displaying increased CapG shuttling in response to EGF. Testing five different potential CapG phosphorylation sites, we found that serine 70 mediates the increase in CapG’s nuclear shuttling triggered by EGF. Thus, repeated FRAP of CapG in the cell nucleus can be used as functional readout of signaling cascades in the same single live breast cancer cell.

## Introduction

Compartmentalization and differential transport of solutes across membranes are critical for the living cell and determine cellular and sub-cellular functions. While the steady-state distribution of proteins can be assessed by immunohistochemistry of fixed cells, the analysis of the dynamic distribution of proteins between cellular compartments requires the use of quantitative live-cell fluorescence microscopy such as fluorescence recovery after photobleaching (FRAP)^1,2^. FRAP was established in the late 1970s to help analyze the ensemble dynamics of molecules in the plasma membrane. Photobleaching of a defined region in a cell creates spatially separated bleached and still fluorescent molecules, thus perturbs the equilibrium of fluorescent molecules and permits the subsequent monitoring of the recovery of the fluorescence equilibrium. FRAP thereby reveals the mobility and net transport of fluorescent molecules.

The gelsolin-related actin-binding protein CapG is the only member of its family that distributes in steady state diffusively and evenly throughout the entire cell, i.e., cell nucleus and cytoplasm^3^. CapG lacks the nuclear export sequence other proteins of its family comprise^4^ and shows no classic nuclear localization signal. A presumed nuclear localization sequence is of uncertain relevance^5^. It has been reported that phosphorylated CapG preferentially localizes to the cell nucleus^6^. Nuclear shuttling of CapG was shown in vitro to require energy and the transport protein importin β^7^. As a capping protein, CapG reversibly blocks the rapidly growing barbed ends of actin filaments^8^. The experimental overexpression or knockout of CapG has been shown to increase and decrease, respectively, the migratory potential of various cell types^9^. CapG was found to be overexpressed in cancer including breast and ovarian cancer^10^. While the function of CapG in the cell nucleus remains unknown, it has been hypothesized that the nuclear CapG fraction is relevant for cell migration and cancer cell invasiveness^7,11^. We previously showed a difference in CapG’s nuclear shuttling in cancer and normal cells as exemplified in the breast cancer cell line MDA-MB-231 and the nearly normal breast epithelial cells MCF-12A^12^. Here, we set out to further characterize the determinants of CapG’s nucleocytoplasmic compartmentalization in the breast cancer cells MDA-MB-231 and resolve changes in CapG shuttling on the single cell level by using fluorescence recovery after photobleaching (FRAP) repeatedly in the same live cell.

## Results

**Fig. 1:**
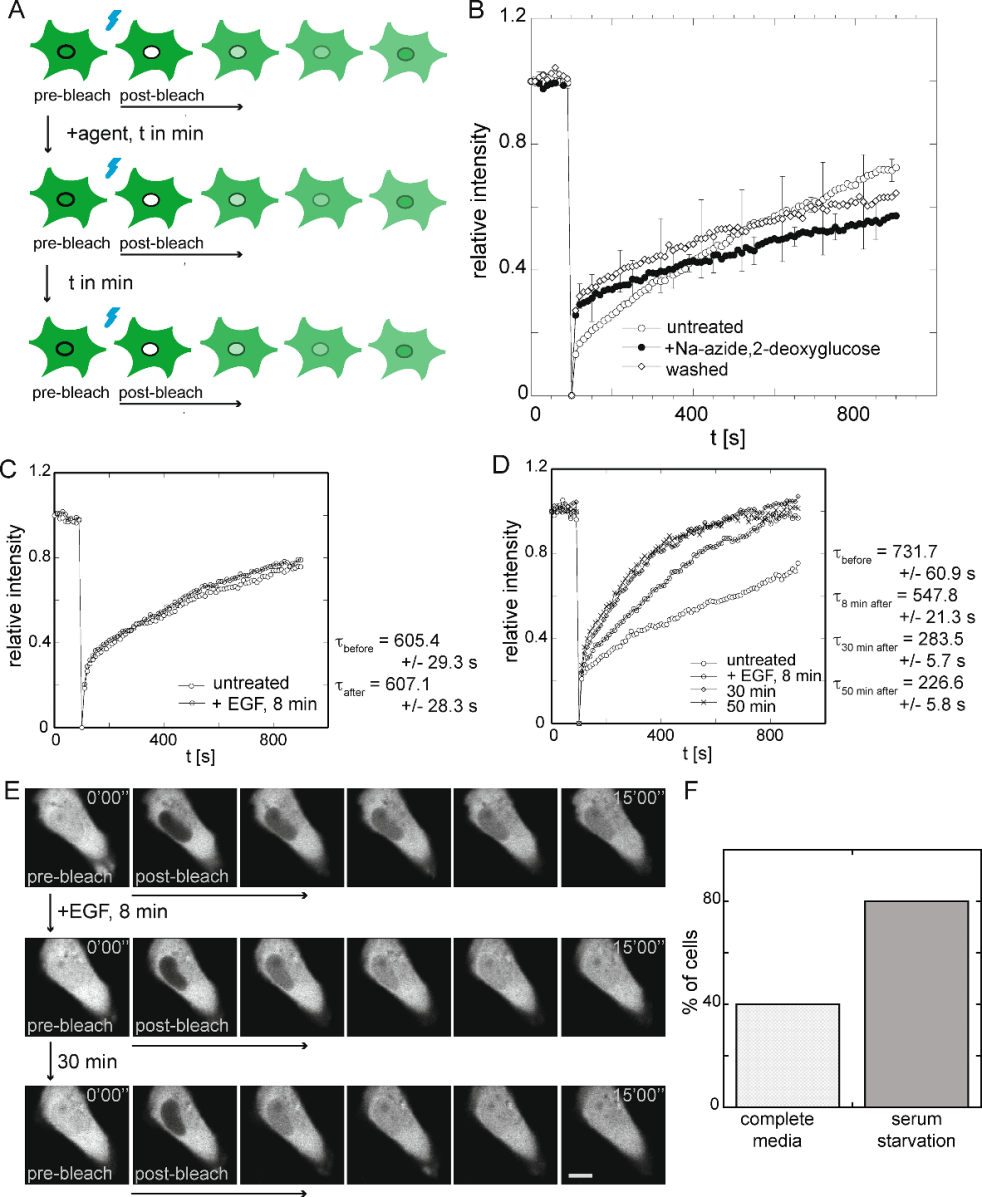
Establishing repeated FRAP of CapG in the cell nucleus of the same live breast cancer as a functional readout for intracellular signaling. (**A**) The schematic illustrates the experimental set-up of repeated FRAP of CapG in the same cell to compare nuclear shuttling before and after the addition of a stimulating or inhibiting agent. (**B**) Fluorescence recovery of CapG-GFP in MDA-MB-231 before, 15 min, and 2 h after ATP-depletion using sodium azide and 2-deoxyglucose. 3 cells were measured. Error bars indicate standard deviations. (**C**) Effect of the addition of EGF on the CapG-GFP nuclear transport in MDA-MB-231 cells. Representative data is shown for the cells (n = 6) that did not show an increase in nuclear CapG-GFP shuttling (Supplemental Fig. 1A). Characteristic recovery times are provided using a single exponential fit. (**D**) Representative data is shown for the cells (n = 4) that showed an increase in nuclear CapG-GFP shuttling (Supplemental Fig. 1B) at 8 and 30 min but leveled off at 50 min. Characteristic recovery times are provided using a single exponential fit. (**E**) MDA-MB-231 cell displaying increased nuclear CapG-GFP import after EGF addition as revealed by the repeated FRAP assay employed here. FRAP experiments performed before EGF addition, as well as 8 min and 30 min after EGF addition. Scale bar 10 um. (**F**): In complete media, 4/10 (40%) MDA-MB-231 cells demonstrated increased CapG-GFP nuclear shuttling, while 8/10 (80%) MDA-MB-231 serum-starved cells displayed increased CapG-GFP nuclear shuttling after the addition of EGF. Single-cell data is shown in Supplemental Figs. 1 and 2, respectively.

Several aspects of the intracellular mobility and compartmentalization of CapG in the living cancer cell which we had characterized previously^12,13^ made us consider using repeated FRAP of the nuclear fraction of CapG as a functional single cell readout for intracellular signaling. (i) CapG displays a large mobile fraction within the cell nucleus of breast cancer cells which results in the homogeneous bleaching of the entire cell nucleus compartment even if a bleaching area is applied that is smaller than the cell nucleus. (ii) The nuclear envelope forms a significant diffusion barrier with CapG nuclear transport taking minutes, i.e., orders of magnitude longer than the bleaching time. (iii) CapG is expressed in fairly high amounts in the breast cancer cell so that repeated bleaching of the cell nucleus compartment and thereby a significant number of CapG-GFP molecules does not pose signal-to-noise problems. And (iv), the MDA-MB-231 breast cancer cells used here proved themselves resilient enough to tolerate repeated photobleaching without obvious signs of phototoxicity over many hours. Therefore, we set out to employ repeated FRAP experiments of CapG-GFP in the cell nucleus of the same live breast cancer cell as shown in Fig. 1A. The main goal was to analyze changes in CapG-GFP’s nuclear shuttling over time in response to external stimuli on the single cell level. After a FRAP experiment was completed, we added an external stimulus, and repeated the FRAP experiment of CapG-GFP in the cell nucleus of the same cell.

To first assess if our repeat FRAP assay could resolve differences in the CapG nuclear transport in the same live cancer cell, we tested its energy dependence which had been reported in in vitro experiments^7^. Using the described repeated FRAP assay, we added 10 mM sodium azide and 6 mM 2-deoxyglucose to achieve ATP-depletion of the cell. After 15 min, the CapG-GFP import into the cell nucleus of MDA-MB-231 cells decreased indicating an ATP-dependent transport process. The decrease in nuclear CapG-GFP import, however, was not irreversible, and cells were not driven into apoptosis, but could be rescued by washing and replacing the media with complete media (Fig. 1B). The third FRAP measurement followed 2 h later and showed recovery and increase in nuclear CapG-GFP shuttling. The viability of the cells was not compromised, neither by the temporary ATP-depletion nor the repeated photobleaching.

Next, we tested if the repeated FRAP assay could be used to analyze the effect of external stimuli on CapG’s nuclear shuttling. The epidermal growth factor (EGF) is a known tumor promoting factor and has been used as chemoattractant in migration assays. EGF receptors are expressed in MDA-MB-231 and known to increase their migratory potential^14^. Since the nuclear fraction of CapG has been hypothesized to be critical for cancer cell invasion^7^, we set out to analyze the effect of EGF on the CapG distribution in the live cell. We hypothesized that EGF may increase nuclear CapG shuttling. After measuring the baseline CapG-GFP transport kinetics in a cell, we added EGF to an end concentration of 200 ng/mL. Because we anticipated a fast non-genomic effect, we repeated the FRAP measurements in the same cell 8 min, 30 min, and 50 min after the addition of EGF. Some MDA-MB-231 cells did not display any change in fluorescence recovery kinetics (Fig. 1C), other cells however did (Fig. 1D). While the recovery curves completely overlapped and showed no difference in their steepness for the cells that did not respond to EGF for cells that did respond to EGF, the steepness of the relative fluorescence intensity curve increased after 8 min and further after 30 min but began to level off at 50 min as shown in Fig. 1D. To extract quantitative information from the recovery curves, we used a single exponential fit. Fitting a single exponential function to the recovery curves shown in Fig. 1C revealed no change in the characteristic recovery time τ, with τ before EGF addition 605.4 ± 29.3 s and 607.1 ± 28.3 s after EGF addition for the cells which showed no difference in the steepness of the recovery curve before and after EGF. This representative cell did not respond to the EGF addition with stimulated CapG nuclear import. In contrast for a cell showing an increased steepness of the recovery curve after the addition of EGF, i.e. stimulated nuclear CapG import, the characteristic recovery time τ increased from 731.7 ± 60.9 s before the addition of EGF to 547.8 ± 21.3 s 8 min after EGF, 283.5 ± 5.7 s at 30 min and 226.6 ± 5.8 s at 50 min after EGF addition (Fig. 1D). Thus, in this representative cell the nuclear transport of CapG-GFP increased 3.2-fold over 50 min (Fig. 1E, Supplemental Movie 1). Analyzing 10 MDA-MB-231 cells, 4/10 (40%) cells showed an increase in CapG-GFP nuclear transport 30 min after the EGF addition, while six cells did not (Fig. 1F, Supplemental Fig. 1). The differing responses of the cancer cells may reflect distinct cell cycle states or indicate that the EGF signaling cascade is not expressed or not active in every cell. To assess a possible cell-cycle dependent EGF response, we serum-starved cells for 20 h and thereby arrested them in the G0 phase. Under these conditions, the addition of EGF triggered in 8/10 (80%) of the analyzed cells an increase in CapG-GFP nuclear shuttling (Fig. 1F, Supplemental Fig. 2). The addition of EGF did not shift the steady-state distribution of CapG-GFP significantly enough to be detected by repeat confocal imaging, neither in MDA-MB-231 cells grown in complete media nor in the serum-starved cells. Confocal images were taken to measure the nucleocytoplasmic fluorescence intensity ratio in steady state before and 30 min after the addition of EGF (Supplemental Fig. 3A ,B) and over time (Supplemental Fig. 3C). No accumulation of CapG-GFP in the cell nucleus was noted in steady state bulk imaging despite the increased nuclear transport as measured by FRAP.

**Fig. 2:**
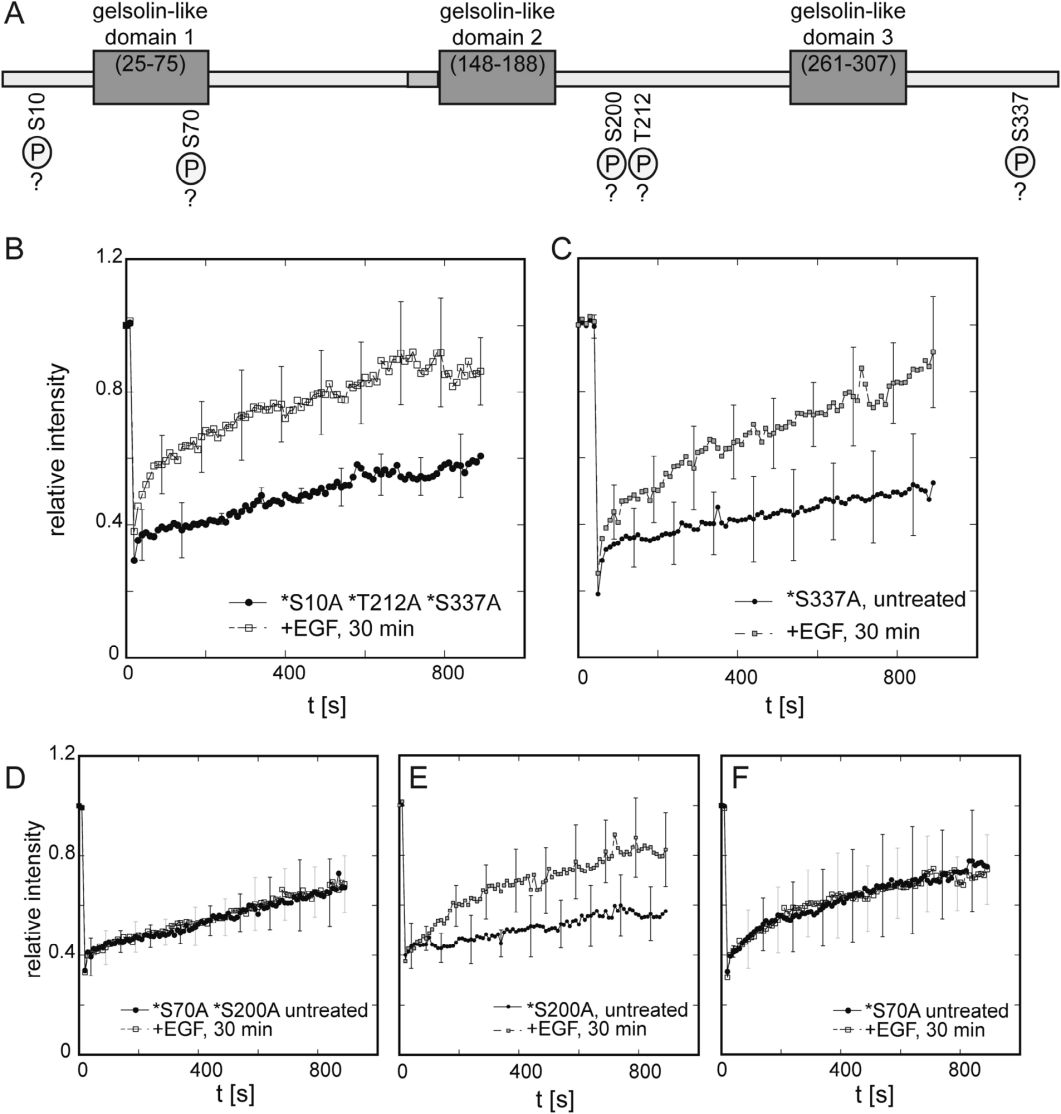
Screening for posttranslational modifications after EGF stimulation using repeated FRAP of CapG in the cell nucleus of the same live breast cancer reveals critical CapG phosphorylation site. (**A**) Schematic of the CapG protein sequence with its 3 gelsolin-like repeats. Potential phosphorylation sites that were analyzed in this study are outlined: S10, S70, S200, T212, and S337. (**B**) Average-value curve of the three analyzed serum-starved MDA-MB-231 cells expressing CapG-GFP with the mutations *S10A, *S337A, and *T212A showed an increase in the steepness of the FRAP recovery curve 30 min after the addition of EGF. Fitting the curve to a single exponential function, the mean recovery time τ is 1,006.7 ± 268.1 s before and 382.2 ± 42.9 s after the addition of EGF. (**C**) Average-value curve of all three analyzed serum-starved MDA-MB-231 cells expressing CapG-GFP with the point mutation *S337A showed an increase in the steepness of the FRAP recovery curve 30 min after the addition of EGF. Fitting the curves to a single exponential function, the mean recovery time τ is 1,368.9 ± 593 s before and 659.5 ± 92.6 s after the addition of EGF. (**D**) 0/8 (0%) serum-starved MDA-MB-231 cells expressing the CapG-GFP mutant *S70A, *S200A displayed increased fluorescence recovery kinetics 30 min after EGF addition. Error bars indicate standard deviations. (**E**) Average-value curve of all three analyzed serum-starved MDA-MB-231 cells expressing CapG-GFP with the point mutation *S200A showed an increase in the steepness of the FRAP recovery curve 30 min after the addition of EGF. Fitting the curves to a single exponential function, the mean recovery time τ is 1,398.9 ± 538 s before and 393.9 ± 139 s after the addition of EGF. (**F**) 0/8 (0%) serum-starved MDA-MB-231 cells expressing the CapG-GFP mutant *S70A displayed increased fluorescence recovery kinetics 30 min after EGF addition. Error bars indicate standard deviations.

**Fig. 3:**
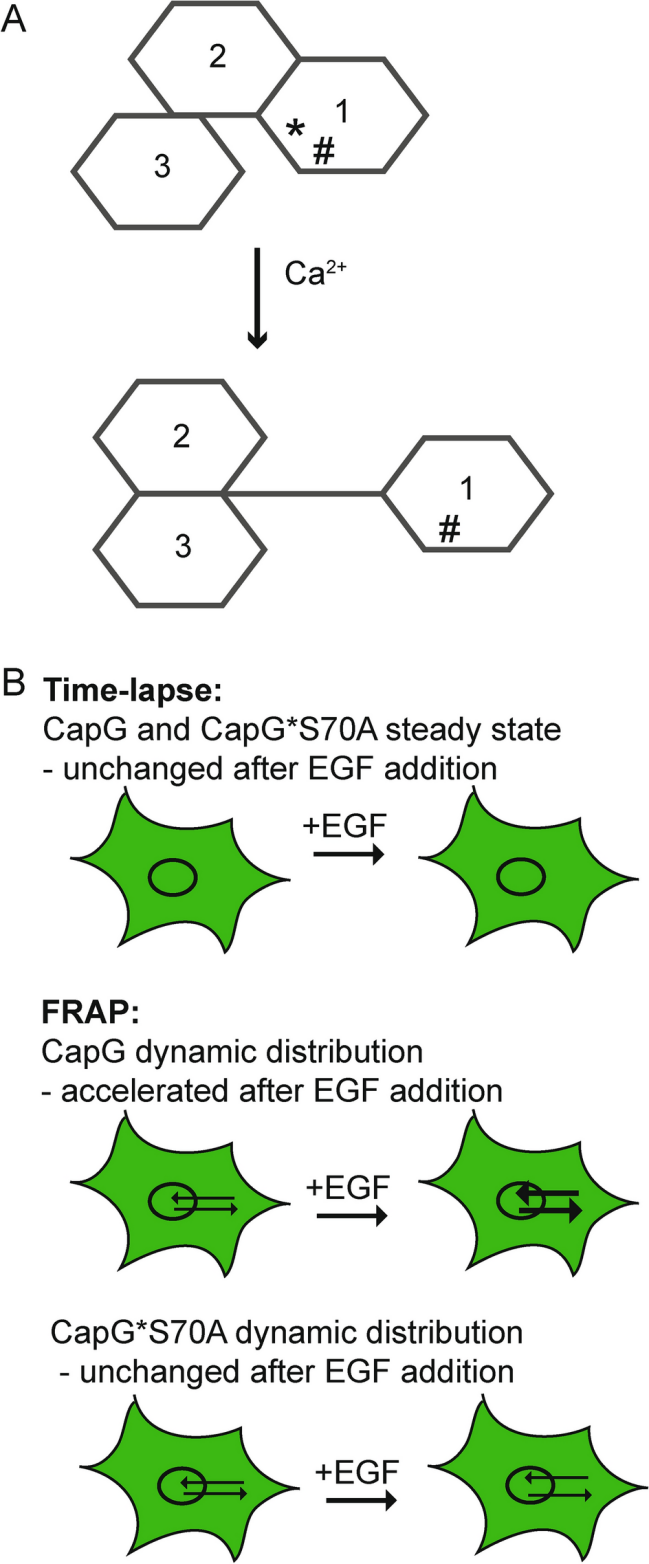
(**A**) Schematic modified from Zhang et al.^21^ showing the hypothetical spatial rearrangement of the Gelsolin-like domains 1–3 (S1-S3) upon Ca-binding. Notably serine S70 is located within the Gelsolin-like domains 1, S1. *Ca-binding site and # phosphorylation site serine S70 in S1. (**B**) Model schematic of CapG distribution and nucleocytoplasmic shuttling in the live cell. While no changes are detectable in steady state, CapG nucleocytoplasmic shuttling is accelerated after EGF addition. Given the lack of significant CapG-GFP accumulation in the cell nucleus, this likely reflects an increase in im- and export of CapG. Serine S70 is the critical CapG phosphorylation site downstream of the EGF signaling cascade.

We then tested which phosphorylation sites in the CapG amino acid sequence are critical for the increase in EGF stimulated CapG nuclear shuttling. To narrow down potential candidates, we used an online available phosphorylation site prediction tool (NetPhos-3.1, DTU Health Tech^15^) (Fig. 2A). In the literature, serines S10 and S337 as well as threonine T212 have been reported to be potential phosphorylation sites^16^. Furthermore, serine S337 has been hypothesized to be located within a protein kinase C site recognition motif^17^. We set out to screen for relevant phosphorylation sites by batching mutations of phosphorylation sites in a single CapG-GFP construct. We hypothesized that if a phosphorylation site is critical for the EGF stimulated nuclear CapG import, mutating this site to an alanine and thereby abrogating the possibility of phosphorylation should prevent EGF-mediated stimulation of CapG nuclear import; in fact, measuring a single cell expressing the CapG-GFP mutant showing stimulated CapG nuclear import would rule out that the mutated phosphorylation site is critical for nuclear CapG import. Conversely, if a phosphorylation site is not critical for the stimulation of CapG nuclear, the measurement of a single cell may show an increase or no change of CapG nuclear import. Even under starvation, only about 80% (and not 100%) of the measured cells showed an increase of CapG nuclear import. Therefore, in this scenario a few cells need to be measured and show no increase. Only when consistently no increase is seen, the mutated phosphorylation site can be assumed to be the critical phosphorylation site. To increase the chance of detecting accelerated CapG-GFP nuclear import after the addition of EGF, all of the following FRAP experiments were performed under serum-starvation as outlined above at least in triplicates. First, we replaced the serines S10 and S337 and threonine T212 with alanines. In cells expressing the triple CapG-GFP mutant *S10A, *T212A, and *S337A, an increase in CapG-GFP nuclear import was noted after the addition of EGF (Fig. 2B). We also tested the amino acid point mutation *S337A in isolation, which again demonstrated stimulation of the CapG-GFP nuclear import by EGF (Fig. 2C). In contrast to the *S10A, *T212A, and *S337A mutants, the CapG mutant *S70A and *S200A did not show any increase in CapG-GFP nuclear transport in the 8 cells analyzed (Fig. 2D, Supplemental Fig. 4A). To confirm whether the serine S200 or serine S70 are the critical phosphorylation sites, they were analyzed in isolation. Cells expressing the CapG-GFP mutant with the *S200A mutation alone demonstrated an increase in CapG-GFP nuclear shuttling (Fig. 2E), while cells expressing the CapG-GFP mutant with the isolated point mutation *S70A did not (Fig. 2F, Supplemental Fig. 4B). Based on the presented repeated FRAP experiments, serine S70 is the critical CapG phosphorylation site whose phosphorylation in response to EGF signaling results in an increase in nuclear transport of CapG (Fig. 3). Again, time lapse imaging did not show any nuclear accumulation or depletion of the mutant CapG-GFP *S70A at steady state and following the addition of EGF (Supplemental Fig. 3D and 3E), only imaging of the nucleocytoplasmic transport dynamics using FRAP was able to reveal changes compared to the wild type CapG.

## Discussion

We showed that the addition of EGF increases nuclear shuttling of CapG in the breast cancer cells MDA-MB-231 within minutes. Thus, the stimulation of the nuclear CapG import stimulation of CapG by EGF is a fast non-genomic effect and likely mediated by phosphorylation. The critical phosphorylation site for this nuclear CapG increase is the serine S70 based on our repeated FRAP experiments, while the potential phosphorylation sites serine S10, S200, S337 and the threonine T212 are not relevant in this context. Serine S70 phosphorylation may increase nuclear CapG shuttling by different mechanisms including (i) increased binding to importin β or other shuttle proteins. ATP depletion has been shown by Miyamoto et al. to impair nucleocytoplasmic transport caused by the loss of RanGTP^18^. van Impe et al. reported that the nuclear transport factor (NTF2) and the GTPase Ran control nuclear import of CapG^19^, another possible mechanism of CapG nuclear import. (ii) Phosphorylation of serine S70 may induce a conformational change that decreases CapG’s hydrodynamic radius and thereby facilitates CapG transport. (iii) It is also possible that mobilization of Calcium from cellular storage sites triggered by EGF signaling^20^ results in Calcium binding to the CapG in Gelsolin-like repeat domain 1 which in turn leads to the extension of the Gelson-like repeat domain 1 away from domain 2 and 3. A crystallographic structure of a mutant CapG is available^21^, based on which Zhang et al. devised a model for this conformational change of CapG. The CapG Gelsolin-like domain 1 (S1) is tightly bound to S2 but only loosely to S3. The authors predicted a Calcium-binding site in S1. Interestingly, the serine S70 is also located in the Gelson-like repeat domain 1 and might be more accessible upon the conformational change upon Calcium binding. Phosphorylation of serine S70 may even help stabilize the extended conformation and thereby facilitate CapG binding to monomeric actin resulting in nuclear co-transport of the actin-binding protein and monomeric actin (Fig. 3A).

The described increase in CapG cell nuclear import triggered by EGF is likely paralleled by an increased CapG export since no significant accumulation of CapG in the cell nucleus was detected on the ensemble or bulk level (Fig. 3B). An increase in nuclear export may be mediated by the co-transport with monomeric actin. Crm1 and XPO-6^22^ have been discussed as relevant export proteins for monomeric actin. Crm1 has been shown to mediate the actin-dependent nuclear export of the serum response factor MAL^23^. Our previous FRAP analyses suggested an only small CapG fraction of 3% that is bound and immobilized in the cell nucleus of MDA-MB-231 cells^13^. The biological meaning of the noted increased nucleocytoplasmic CapG shuttling is thus far uncertain and relates to the open question of CapG’s function in the cell nucleus. Various binding partners in the cell nucleus have been suggested in the literature and include steroid receptors^5,24^, NF-κB^25^, and/or nuclear actin. The high abundance of the freely diffusive CapG in the cell nucleus may serve as a pool of capping proteins for transient need, as it were ready on demand. Calculations of CapG in the cytoplasm showed a 5 to tenfold abundance relative to the plus ends of actin filaments in macrophages^26^. It is also feasible that CapG co-shuttles and transports cargo into the cell nucleus and that such cargo in turn exerts the biological function in the cell nucleus. Studies addressing these questions are ongoing.

Repeated FRAP of CapG in the cancer cell nucleus permits assessment of the functionality of signaling cascades on the single cell level provided those signaling cascades affect CapG shuttling. A functional single cell assay seems to be especially important since upstream receptors of signaling cascades may not be expressed in every cell or may not be biologically active even if expressed which has been reported for the EGF receptor in breast cancer cells^27^. Ensemble analyses may average out and thus miss single-cell behavior. In our hands, repeated FRAP experiments in the same live cell did not trigger any apparent cell toxicity and cells could be imaged within any signs of compromise over many hours. EGFR is expressed in the triple negative MDA-MB-231 cells, i.e., MDA-MB-231 cells are negative for Her2, and the hormone receptors ER (estrogen receptor), and PR (progesterone receptor). Price et al. showed that EGFR signaling in MDA-MB-231 may result in increased migration rather than increased proliferation^14^. Especially the nuclear fraction of CapG has been hypothesized to be critical for cancer cell invasion^7^.

In summary, we showed that repeated FRAP of CapG in the cell nucleus is feasible and can resolve changes in nuclear shuttling which are inaccessible to repeated static imaging. Repeated FRAP of CapG in the cell nucleus is a suitable functional assay to assess downstream effects of signaling cascades in the single living cancer cell and demonstrated that CapG’s nuclear shuttling increased within minutes after EGF stimulation and depends on the phosphorylation of serine S70.

## Material and methods

### Plasmids and transfections

We used the plasmid CapG-eGFP (in a clontech N1 vector) as previously published^12^. For simplicity, we call the construct CapG-GFP throughout the manuscript. Site-directed mutagenesis was performed using the QuikChange Kit from Agilent according to the manufacturer’s protocol. The serines S10, S70, S200, S337 and the threonine at T212 were replaced by alanines. The following primers were used 5’-ATT CCC CAG AGT GGC GCT CCA TTC CAG GCT CAG T-3’ for *S10A, 5’- CCA GCA GTC AGC CCG GGA TGA GCA -3’ for *S70A, 5’-GCC ATC CGG GAC GCG GAG CGA CAG GGC AA-3’ for *S200A, 5’-CAG GTG GAG ATT GTC GCT GAT GGG GAG GAG CCT GCT-3’ for *T212A, 5’-TCT GCC TCA GGG CCG TGA GGC TCC CAT CTT CAA GCA ATT T-3’ for *S337A, respectively. MDA-MB-231 cells were transfected with the CapG-GFP construct and its mutants using Transfectin (Biorad, Hercules, CA) according to the manufacturer’s protocol.

### Cell culture

The MDA-MB-231 cell line, a human breast cancer cell line, was obtained from ATCC (HTB-26) and cultured in DMEM without phenol red (Gibco cat# 11,054,020) and supplemented with 10% fetal bovine serum (FBS) and 2 mM glutamine.

### FRAP and confocal microscopy

The Zeiss laser-scanning confocal microscope LSM710 with a 25-mW Argon ion laser and a 62 × 1.4 N.A. objective was used to perform the photobleaching experiments. The FRAP experiment was performed by exposing defined regions of cells to 100% laser intensity for 20 iterations. Images were taken with laser intensity set to 0.1% as indicated by the control software.

For quantitative analysis, regions were defined on the acquired images delineating the bleached nucleus, the cytoplasm and the extracellular space. By integrating over all pixels using the image processing software Fiji (NIH, Bethesda), the fluorescence intensity was determined for each time point in each region. The determined mean gray values were background subtracted, corrected for acquisition photobleaching, laser intensity fluctuations and loss of fluorescence due to the bleaching event. The data were normalized to the initial fluorescence intensity. The following equation for the normalized fluorescence was applied:

$$F\left(t\right)= \frac{Roi\left(t\right)-BG(t)}{Tot\left(t\right)-BG(t)} \times \frac{Tot\left({t}_{0}\right)-BG({t}_{0})}{Roi\left({t}_{0}\right)-BG({t}_{0})}$$

where Roi(t) denotes the fluorescence intensity at time t in the region of interest, i.e., the cell nucleus, Tot(t) denotes the fluorescence intensity in the cytoplasm at each time point and BG(t) the extracellular background fluorescence in each time point^2,12^. The normalized relative fluorescence intensity curve was fit with the single exponential function $F \left(t\right)=1-[a-b\left(1-{e}^{-\lambda t}\right)]$, where $a$ is the fraction of fluorescence intensity initially bleached, $b$ the fraction that recovered after the time $t$ with the rate $\lambda$. The recovery time τ is the reciprocal of the recovery rate λ. An increase in nuclear CapG import was stated if the slope of the recovery curve was steeper after the EGF addition compared to before (qualitative criterion) and/ or the single exponential fit showed a decrease in the characteristic recovery time τ by at least 20% after 30 min (quantitative criterion).

FRAP experiments were performed at 37 ℃ in Hepes-buffered medium (pH 7.3 with 20 mM Hepes) 20 h after transfection. For serum starvation, cells were washed with Hanks balanced salt solution, and DMEM media with 0.1% fetal bovine serum and 2 mM Glutamine were added to the cells. Cells were incubated in this media 20 h before the experiments. Epidermal growth factor (EGF, Sigma) was reconstituted as per manufacturer’s instructions. A 100 ug/ mL stock solution was aliquoted and stored at −80 ℃. EGF was added for the experiments to the cell culture media in an end concentration of 200 ng/mL.

## Supplementary Information

Supplementary Video 1.

Supplementary Information 1.

## Data Availability

The datasets generated and analyzed during the current study are available from the corresponding author on reasonable request.
