# Supplementary material for: Repeated FRAP of the actin-binding protein CapG in the cell nucleus—a functional assay for EGF signaling in the single live breast cancer cell
Source: Sci Rep. 2024 Oct 5;14:23159. doi: 10.1038/s41598-024-73887-7 (PMC11455965; doi:10.1038/s41598-024-73887-7)
Supplement: Supplementary file 2 — Supplementary Information 1. [file 41598_2024_73887_MOESM2_ESM.pdf]

Supplemental Figures and Movies

Supplemental Figure 1: 6/10 MDA-MB-231 cells did not show an increase in CapG-GFP nuclear transport 30 min after the EGF addition (**1A**), while 4/10 (40%) cells did (**1B**). All analyzed cells are shown here.

Supplemental Figure 2: After 20 h serum starvation, only 2/10 cells did not show an increase in CapG-GFP nuclear transport 30 min after the EGF addition (**2A**), while the addition of EGF triggered in 8/10 (80%) of the analyzed cells an increase in CapG-GFP nuclear shuttling (**2B**). All analyzed cells are shown here.

Supplemental Figure 3A: The mean nucleocytoplasmic intensity ratio of CapG-GFP, i.e., steady-state distribution of CapG-GFP, in single MDA-MB-231 cells grown in complete media and under serum starvation did not show a significant difference. **Supplemental Figure 3B:** Also, 30 min after stimulation with EGF, there was no difference in the nucleocytoplasmic intensity ratio of CapG-GFP detectable. **Supplemental Figure 3C:** Time lapse imaging every 3 min of serum-starved CapG-GFP expressing cells immediately before and following the addition of EGF showed no change over time. **Supplemental Figure 3D:** The mean nucleocytoplasmic intensity ratio of CapG-GFP *S70A in complete media and under serum starvation did not show a significant difference. **Supplemental Figure 3E:** Time lapse images every 3 min in serum-starved CapG-GFP *S70A expressing cells immediately before and following the addition of EGF showed no change over time.

Supplemental Figure 4A: All 8 analyzed MDA-MB-231 cells expressing CapG-GFP with the mutations *S70A and *S200A did not show any increase in the slope of the FRAP recovery curve 30 min after the addition of EGF.

Supplemental Figure 4B: All 8 analyzed MDA-MB-231 cells expressing CapG-GFP with the point mutation *S70A did not show any increase in the slope of the FRAP recovery curve 30 min after the addition of EGF.

Supplemental Movie: MDA-MB-231 cell displays increased nuclear CapG-GFP import after EGF addition as revealed by the repeat FRAP assay of the same cell. Upper panel displays MDA-MB-231 cell before, lower panel the same cell 30 min after EGF addition.

A MDA-MB 231 in complete media

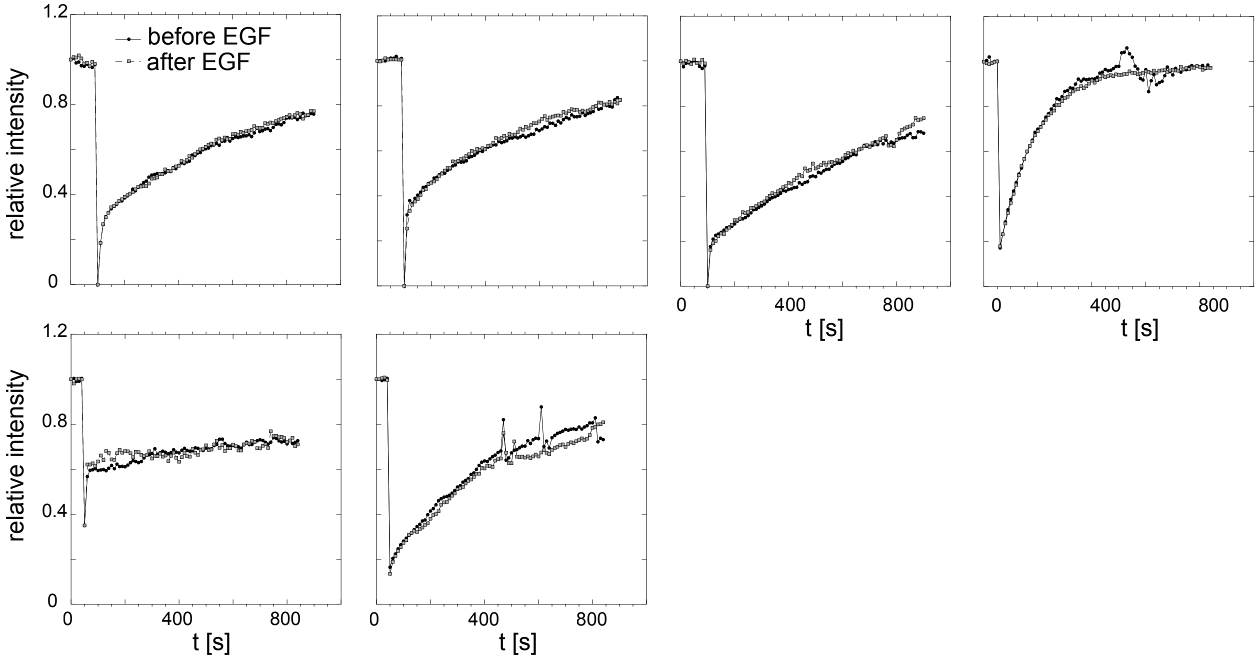

B

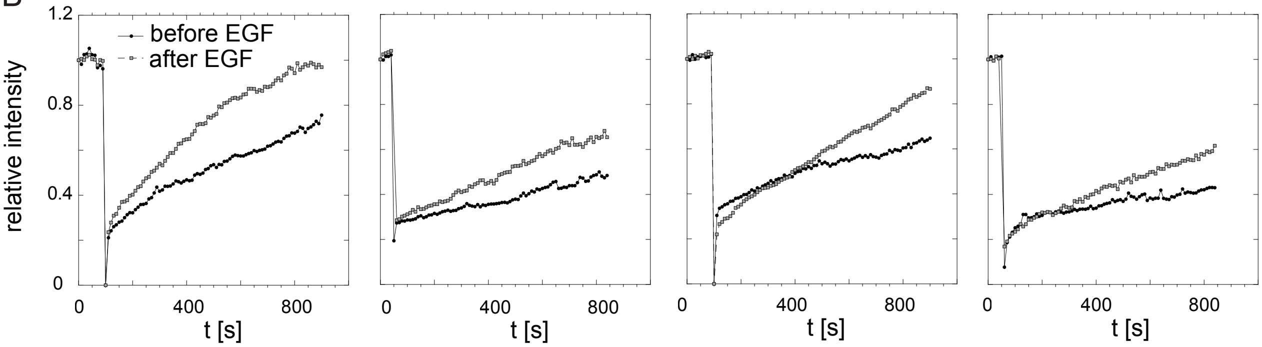

Fernandez et al. Supplemental Fig. 1

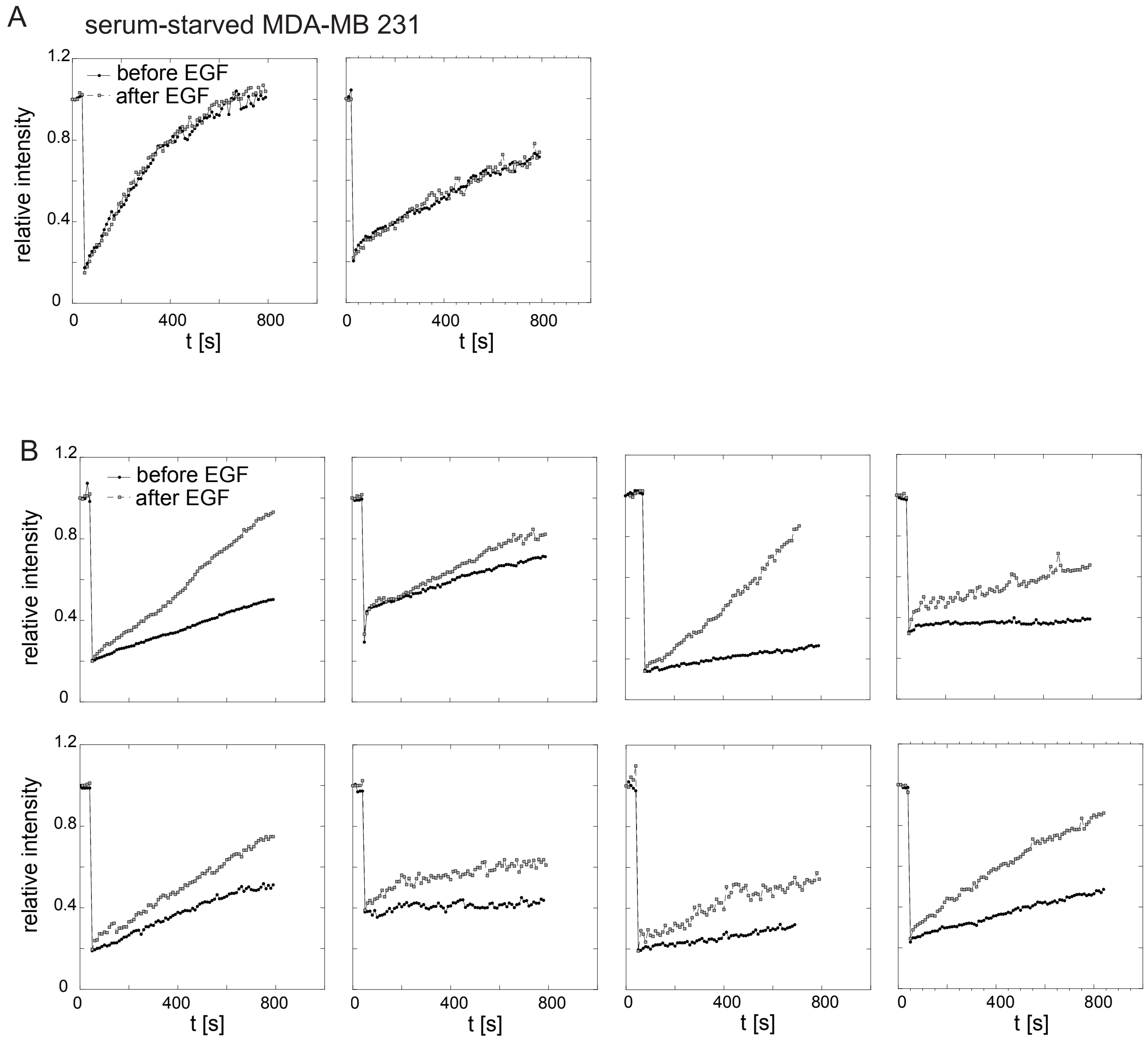

Fernandez et al. Supplemental Fig. 2

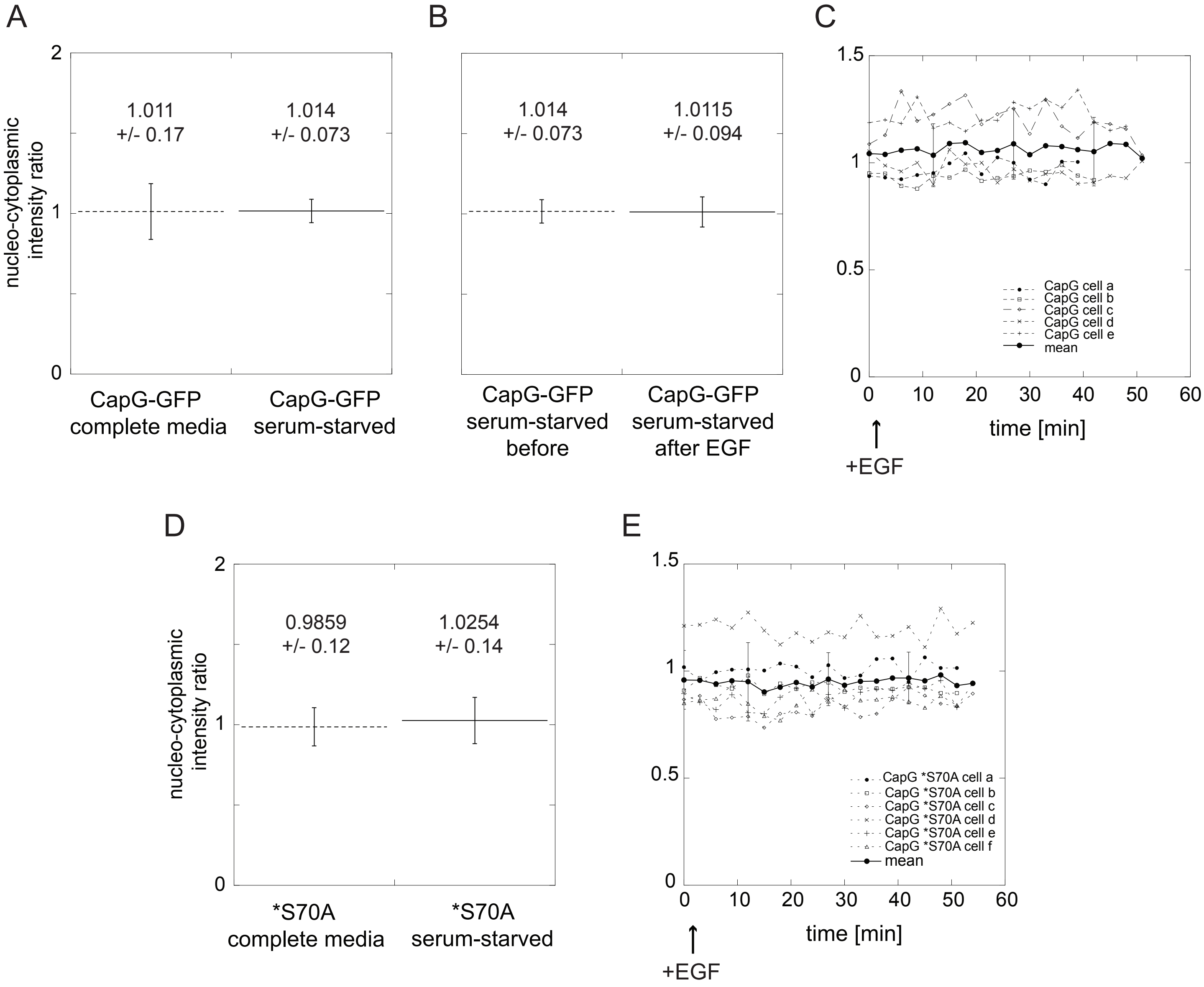

Fernandez at al. Supplemental Fig. 3

A CapG-GFP *S200A *S70

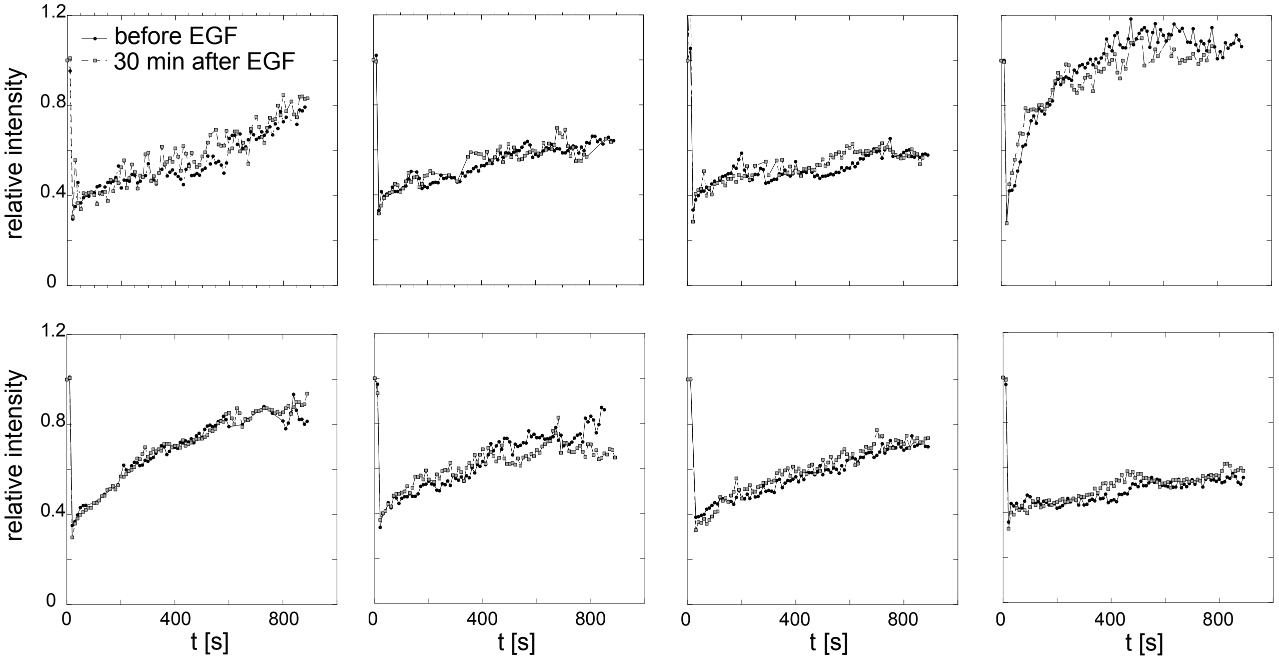

B CapG-GFP *S70A

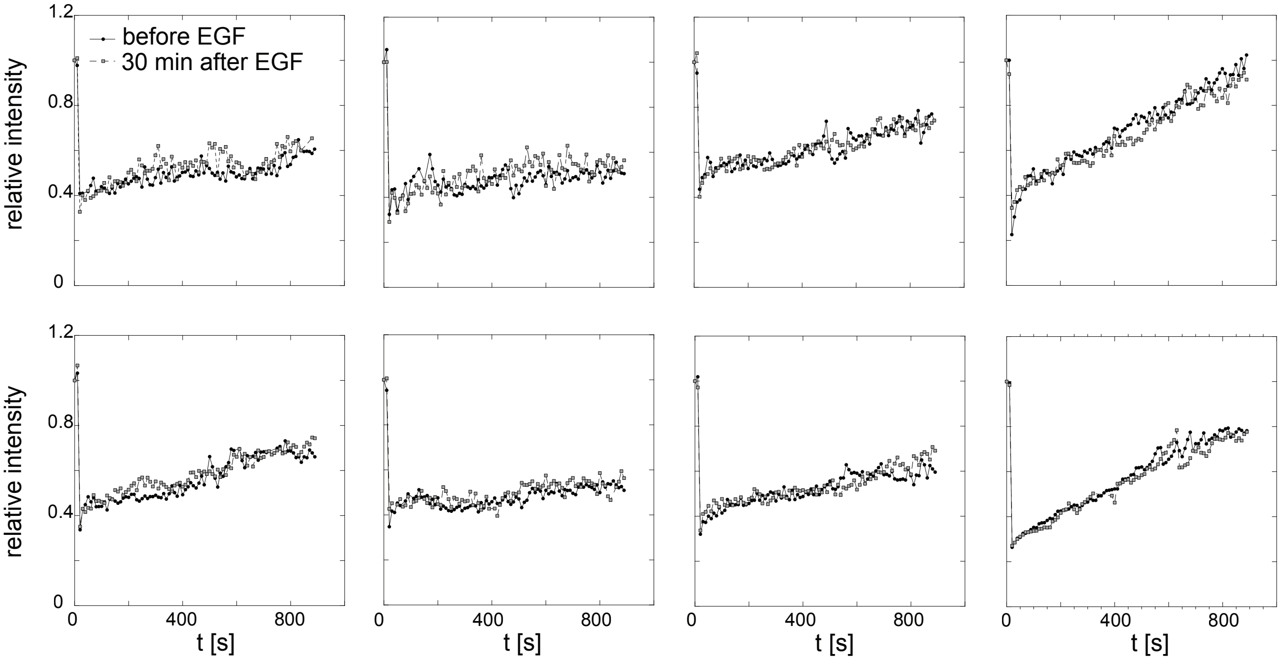

Fernandez at al. Supplemental Fig. 4
